# Clonal Relationship and Resistance Profiles Among ESBL-Producing *Escherichia coli*

**DOI:** 10.3389/fcimb.2021.560622

**Published:** 2021-06-23

**Authors:** Alireza Dolatyar Dehkharghani, Setareh Haghighat, Marjan Rahnamaye Farzami, Mohammad Rahbar, Masoumeh Douraghi

**Affiliations:** ^1^ Department of Microbiology, Faculty of Advanced Sciences and Technology, Tehran Medical Sciences, Islamic Azad University, Tehran, Iran; ^2^ Department of Microbiology, Research Center of Reference Health Laboratory, Ministry of Health and Medical Education, Tehran, Iran; ^3^ Division of Microbiology, Department of Pathobiology, School of Public Health, Tehran University of Medical Sciences, Tehran, Iran

**Keywords:** *E. coli*, AmpC, molecular typing, resistance, gene

## Abstract

AmpC β-lactamases hydrolyze all β-lactams except cefepime and carbapenems. The study of AmpC-producing *E. coli* has high priority for the infection control committee. This research is aimed to investigate the resistant urinary AmpC-generating *E. coli* isolates and identify their genetic variety. Some 230 *E. coli* isolates from patients suffering urinary tract infection symptoms were studied in 2017–2018 to assess their susceptibility toward antimicrobial agents. AmpC gene was evaluated by PCR and molecular typing using the 10-loci MLVA method. MLVA images were examined by BioNumerics 6.6 software through the use of the UPGMA algorithms. Thirty-eight AmpC-generating *E. coli* isolates were detected. The most abundant determinant was *bla_CIT_* and *bla_EBC_*, *bla_FOX_*, and *bla_DHA_* had the next ranks, respectively. Six major clusters and a singleton were identified by MLVA. AmpC beta-lactamases in urinary isolates of *E. coli* in the hospital under study and high rate of additional resistance to gentamicin, cotrimoxazole and ciprofloxacin. The most frequent gene determinant of AmpC beta-lactamase was *bla_CIT_* and vary depending on time and geographical location.

## Background

*Escherichia coli* is the first infectious agent in the development of urinary tract infection (UTI) (Connie et al., 2014) accounting for more than 85% UTI cases (Queenan and Bush, 2007). One hundred and fifty million cases of urinary tract infection are annually reported throughout the globe (Dallenne et al., 2010). It is 30 times more prevalent among females and almost 60% of women have the experience of UTI at least once in their lives (Dallenne et al., 2010). Antimicrobial agents can greatly contribute to the clinical control of UTI. Antimicrobial resistance, in particular, multidrug-resistant Gram-negative bacilli, has posed a remarkable challenge for the clinical settings (Saffar et al., 2016). Diverse drug resistance mechanisms have been introduced in Gram-negative bacteria among which, extended-spectrum β-lactamases (ESBL) production, AmpC β-lactamase generation, efflux, and porin deficiency can be mentioned. AmpC β-lactamases and ESBLs can be identified in the clinical laboratories. AmpC β-lactamases are capable of causing resistance to both narrow- and broad-spectrum cephalosporins, β-lactam/β-lactamase inhibitor combinations, and aztreonam (Xu et al., 2015). They can be chromosomally modified or plasmid-mediated. The plasmid-mediated AmpC β-lactamases can hydrolyze the entire β-lactam antibiotics, except Cefepime and carbapenems. The plasmid-mediated AmpC genes are originated from inducible chromosomal genes mobilized in different organisms. *blaACC*, *blaFOX*, *blaMOX*, *blaDHA*, *blabla*, *blaCIT*, and *blaEBC* are among the well-known gene determinants (Turnidge, 1998; Nikaido and Pagès, 2012; Nakano et al., 2017). AmpC detection plays a crucial role in the clinical management of infections as it can offer epidemiological data. Despite specific tests to detect beta-lactamases such as using inhibitor based test with phenylboronic acid (PBA) or cloxacillin, there is no mandatory guideline for their diagnosis in clinical laboratories (Hopkins et al., 2006). Considering the scarce epidemiological data, this study is aimed to assess the frequency of *blaACC*, *blaFOX*, *blaMOX*, *blaDHA*, *blaCIT*, and *blaEBC* genes in the urinary AmpC-generating *E. coli* strains collected from the patients with UTI. The second goal is the determination of genetic diversity in the AmpC-generating strains.

## Material and Methods

During 2017–2018, we collected 230 isolates of AmpC and ESBL-producing *E. coli* from patients with UTI symptoms who were hospitalized at Milad hospital. The Milad hospital is a 1,000-bed tertiary care hospital, affiliated with the social assurance organization. The isolates were identified using standard biochemical tests. Antimicrobial susceptibility testing was performed by the E-test (MIC method). All isolates were stored at −80°C in tryptic soy broth containing 15% glycerol for future molecular studies.

### Antimicrobial Susceptibility Tests

Antimicrobial susceptibility tests were conducted on *E. coli* isolates according to the E-test method (***Liofilchem*** ^®^ MIC ***Test Strips***). The MICs (minimum inhibitory concentration) of eight antibiotics (ceftazidime, ceftriaxone, gentamicin, meropenem, ciprofloxacin, piperacillin/tazobactam, and trimethoprim/sulfamethoxazole) against the *E. coli* isolates were investigated. The results were recorded based on M100-CLSI 2019 guideline and categorized into three groups: resistant, intermediate, and susceptible.

### Detection of AmpC-Positive Isolates

The screening tests were carried out on isolated *E. coli* strains to detect AmpC-Positive Isolates by the use of cefoxitin (30 µg) disk. Subsequently, the suspected strains were further verified by an AmpC detection set (*MAST DISCS*™ID, UK). AmpC detection set has been validated for identification of AmpC β-lactamase. *E. coli* ATCC 25922 and *Enterobacter cloacae* ATCC 13047 served as negative and positive controls, respectively, for the production of AmpC β-lactamase. The findings of Antimicrobial susceptibility testing (AST) and AmpC detection tests were entered in WHONET software.

### Detection of ESBL-Positive Isolates

The ESBL-resistance *E. coli* isolates were confirmed using the combination disc based on CLSI guidelines (CLSI 2019). In a typical procedure, a ceftazidime disc (CAZ) (30 μg) alone and one in combination with clavulanic acid (CAC) (30/10 μg) were applied to confirm the isolates. Both discs were placed on a Mueller Hinton Agar (*MHA*) plate followed by overnight incubation at 37°C. The distance between the centers of the discs was set to 20 mm. ESBL-positive involved A ≥5 mm increase in zone diameter of either antimicrobial agents combined with clavulanic acid as compared to their zone upon the use of antimicrobial agents alone. *K. pneumoniae* ATCC 700603 and *E. coli* ATCC 25922 were utilized as ESBL-positive and negative controls, respectively.

### PCR Reactions for AmpC-Producing Genes

Genomic DNA extraction was carried out by High Pure PCR Template Preparation kit (Roche, Germany). The detection of AmpC- β-lactamase and ESBL genes in urinary *E. coli* isolates were conducted by PCR employing the specific oligonucleotide primers as presented in **Table 1**. PCR amplification was achieved in a Peqlab PCR thermal cycler with the PCR Master Mix (Ampliqon Inc., Denmark) following the manufacturer’s guidelines. The PCR reactions were carried out on 25 µl volume. The master mix included 12 µl Master Mix Red (Ampliqon Inc., Denmark) and 1 µl target DNA. Forward and reverse primers were added as mentioned in **Table 1**. The reaction volume was increased to 25 μl by adding sterile distilled water. The addition of forward and reverse primers and temperature profile was performed by the Queenan’s protocol (Mansouri et al., 2009).

**Table 1:** Primer sequences for AmpC genes.

Target gene	Primer		Tm °C	Product size	Ref.
FOX	F	AAC ATG GGG TAT CAG GGA GAT G	56	190	(Mansouri et al., 2009)
	R	CAA AGC GCG TAA CCG GAT TGG	59.7		
MOX	F	GCT GCT CAA GGA GCA CAG GAT	59.6	520	
	R	CAC ATT GAC ATA GGT GTG GTG C	56.6		
EBC	F	TCG GTA AAG CCG ATG TTG CGG	60.5	302	
	R	CTT CCA CTG CGG CTG CCA GTT	62.7		
ACC	F	AAC AGC CTC AGC AGC CGG TTA	61.2	346	
	R	TTC GCC GCA ATC ATC CCT AGC	60.1		
DHA	F	AAC TTT CAC AGG TGT GCT GGG T	59.3	405	
	R	CCG TAC GCA TAC TGG CTT TGC	59.2		
CIT	F	TGG CCA GAA CTG ACA GGC AAA	59.3	462	
	R	TTT CTC CTG AAC GTG GCT GGC	60.1		

### Multi-Locus Variable Number of Tandem Repeats Analysis (MLVA)

The complete *E. coli* DNA was provided from overnight cultured samples utilizing High Pure PCR Template Preparation Kit (Roche, Germany). *E. coli* MLVA was conducted by the seven tandem sequence repeats (CVN001, CVN002, CVN003, CVN004, CVN007, CVN014, CVN015) according to Lindstedt et al. (Helmy and Wasfi, 2014). Moreover, CVN016, CVN017, and a regularly-interspersed short palindromic repeat (CCR001), which have been reported to enhance the discriminatory power of the MLVA, were included with 10-loci *E. coli* MLVA (Nadjar et al., 2000). **Table 2** lists the applied primers. Repeats were amplified by PCR and assessed on 3% agarose. Here, the size of PCR products was simply assayed on agarose gel with no complication using 100 bp and 20 bp size markers (Bio-Rad Laboratories Inc.).

**Table 2:** Primer sequences for MLVA technique.

Target locus		Primer sequence	Tm °C	Reference
**CVN001**	F	AACCGGCTGGGGCGAATCC	62.4	(Helmy and Wasfi, 2014)
	R	GGCGGCGGTGTCAGCAAATC	62	
**CVN002**	F	AACCGTTATGAARGRAAGTCCT	53.8	
	R	TCGCCCAGTAAGTATGAAATC	52.4	
**CVN003**	F	AAAAATCCGGATGAGWTGGTC	54.9	
	R	TTGCGTTGTCAGTAATTTGTTCAG	55.3	
**CVN004**	F	MGCTGCGGCRCTGAAGAAGA	63.2	
	R	CCCGGCAGGCGAAGCATTGT	63.2	
**CVN007**	F	ACCGTGGCTCCAGYTGATTTC	57.8	
	R	ACCAGTGTTGCGCCCAGTGTC	61.1	
**CVN014**	F	TCCCCGCAATCAGCAAMACAAAGA	62.6	
	R	GCAGCRGGGACAACGGAAGC	63.8	
**CVN015**	F	TAGGCATAGCGCACAGACAGATAA	58.2	
	R	GTACCGCCGAACTTCAACACTC	58.6	
**CVN016**	F	GCTGCAGGAGAATGGGATGGTTTT	60.1	(Nadjar et al., 2000)
	R	GGTGAGGTGTCCGAGTGGCTGAAG	63.4	
**CVN017**	F	GCAATCACCGCCGCAATCTGTT	61.6	
	R	CGCCGCCGAAGCAAATCTC	59.8	
**CCR001**	F	CTCAGGGAAAAGGGAAGACACTAC	57	
	R	TTGCACTGAACACCGAATACG	56.2	

The following formula was employed to determine the VNTR (Variable-Number Tandem Repeat) repeat numbers of each locus; ((NPS − OF)/RL, in which PS = product size, OF = offset region (region of repeat-free sequence) and RL = length of one repeat unit (Tan et al., 2009).

## Results

Among 230 urinary *E. coli* isolates, 87 samples were from the male cases, while the remaining 142 were sampled from female subjects. The patients’ age varied one to 93 years (average: 58.81 ± 25.07).

### Phenotypic Detection of AmpC- and ESBL-Producing Isolates

Thirty-eight AmpC-producing *E. coli* isolates were detected by cefoxitin (30 µg) among which, 35 isolates were confirmed by the AmpC detecting test (MASTDISCS™ID, UK). Among these 35 AmpC-producing *E. coli* isolates, nineteen cases were AmpC and ESBL coproducer; while sixteen isolates were AmpC-positive and non-ESBL.

About 40% (14/35) of AmpC-producing isolates showed resistance toward gentamicin. Some 65.7% (23/35) and 74.2% (26/35) of the studied isolates were resistant against trimethoprim-sulfamethoxazole and ciprofloxacin, respectively.

**Figure 1:**
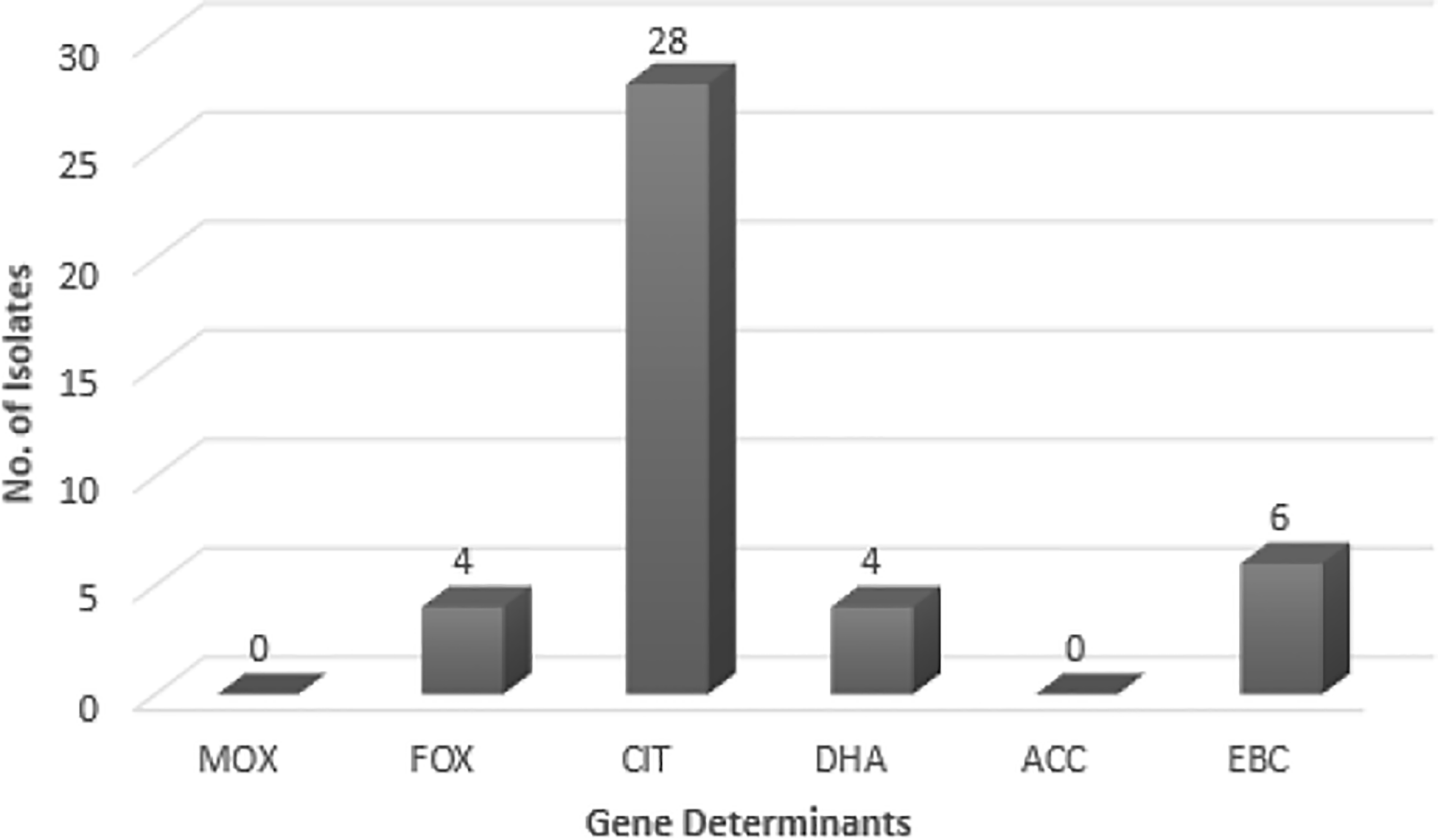
The frequency of *AmpC* genes in *E. coli* isolates. MOX, Active on Moxalactam; *FOX*, Active on Cefoxitin; *CIT*, Firstly isolated from *Citrobacter freundii*; *DHA*, Firstly isolated at Dhahran Hospitals in Saudi Arabia; *ACC*, Ambler class C; *EBC*, Isolated from *Enterobacter cloacae*.

As suggested by **Figure 1**, no *bla_MOX_* and *bla_ECC_* genes were detected in uropathogenic *E. coli* isolates, while, 73.6% of the tested samples contained *bla_CIT_*. *bla_FOX_* (*bla_FOX-1_* to *bla_FOX-5_*), *bla_DHA_* (*bla_DH7_* and *bla_DHA-2_*) and *bla_EBC_* were detected in 10.5, 10.5 and 15.8% of AmpC-producing *E. coli* isolates, respectively. Four isolates had both *bla_CIT_* and *bla_EBC_*. *bla_CIT_* and *bla_FOX_* were simultaneously present in four isolates. Two isolates also possessed both *bla_CIT_* and *bla_DHA_*. The *bla_CIT_* group of AmpC β-lactamases prevalently encompassed *bla_LAT-1_* to *blabla*, *bla_BIL-1_*, *bla_CMY-2_* to *bla_CMY-7_*, *bla_CMY-12_* to *bla_CMY-18_*, and *bla_CMY-21_* to *bla_CMY-23_* (Dallenne et al., 2010). AmpC and ESBL-coproducing isolates had *bla_CIT_*, and four isolates having *bla_CIT_* and *bla_EBC_*, were AmpC and ESBL-coproducer.

### Typing of AmpC-Producing *E. coli* Isolates by MLVA

**Figure 2:**
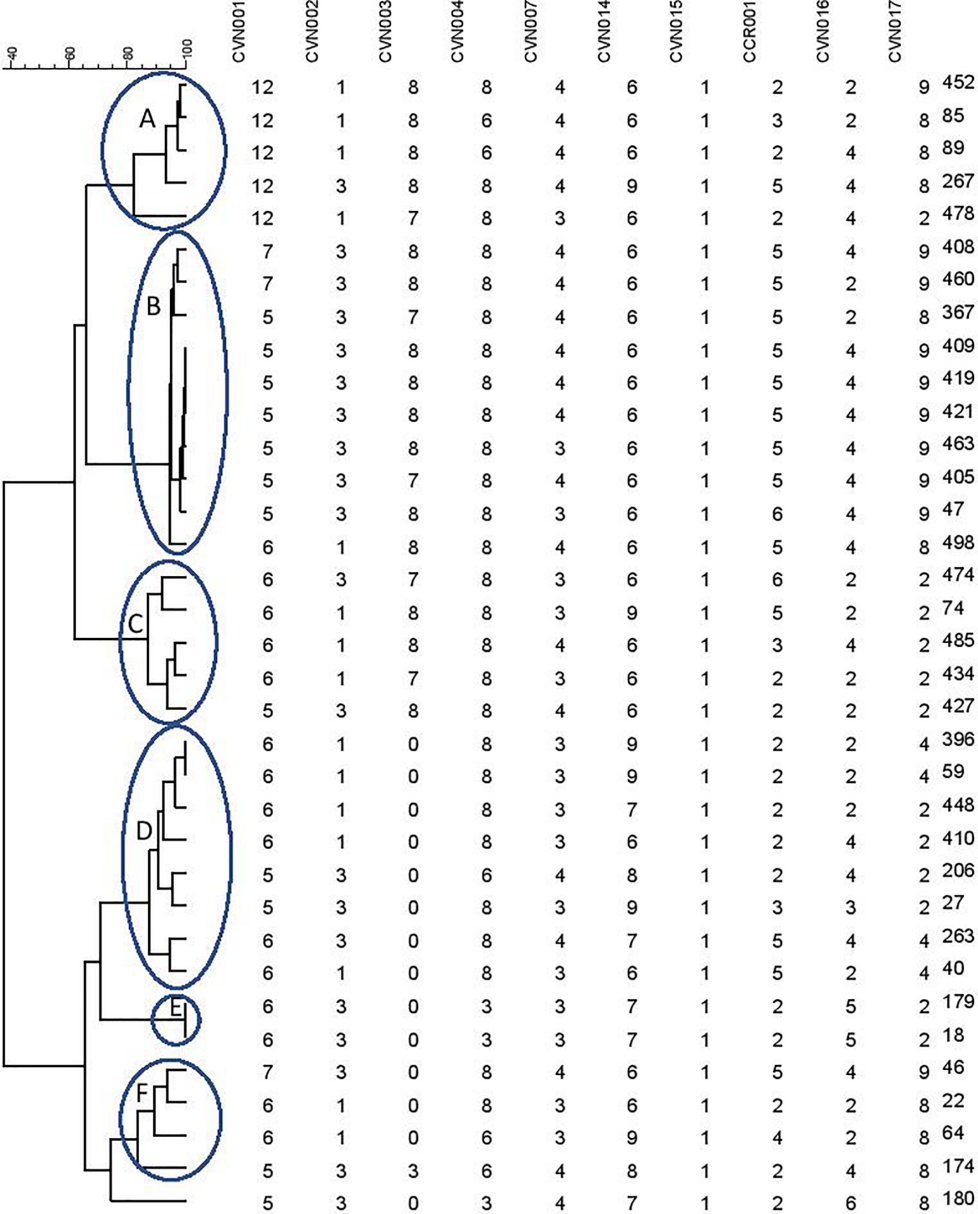
Clustering results of b-lactamase-producing *E. coli* using 10-loci MLVA (**A–F** clusters) differentiated UPGMA with the categorical coefficient of similarity.

A dendrogram was formed based on 10-loci using BioNumerics software ver.6.6 and the 31 MLVA types created six major clusters (labeled A to F) by 10-loci based according to their genetic similarity (cutoff* of *80%). Moreover, a singleton was detected in AmpC-producing *E. coli* isolate sampled from UTI patients. In the present research, 30 distinct genotypes were categorized by 10-loci MLVA typing of 35 AmpC-producing *E. coli* isolates (**Figure 2**).

As mentioned in **Table 3**, the resistance profile exhibited no significant relationship with MLVA pattern and the AmpC or ESBL production ability of *E. coli*.

**Table 3:** Characterization of AmpC Positive *E. coli* Isolates.

Isolate Code	MLVAProfile	Gene profile	ESBL	CRO	CIP	SXT	TZP	MEM	AMC	GEN	CAZ
452	A	*CIT*	*+*	R	S	R	R	S	R	R	R
85	A	*CIT*, *FOX*	*+*	R	R	R	R	S	R	S	R
89	A	*CIT*, *EBC*	*+*	R	R	R	R	S	R	S	R
267	A	*CIT*, *EBC*	*+*	R	R	R	R	S	R	R	R
478	A	*EBC*	*+*	R	R	R	I	S	R	R	R
408	B	*CIT*, *FOX*	*+*	R	R	R	R	S	R	S	R
460	B	*CIT*	*+*	R	R	S	I	R	R	R	R
367	B	*CIT*, *EBC*	*+*	R	R	R	I	I	R	R	R
409	B	*CIT*, *EBC*	*+*	R	R	R	R	S	R	R	R
419	B	*CIT*, *FOX*	*+*	R	R	R	R	S	R	R	R
421	B	*CIT*	*+*	S	S	S	S	S	R	S	S
463	B	*CIT*, *FOX*	*+*	R	S	R	S	S	R	R	R
405	B	*CIT*	*+*	R	R	S	S	S	R	R	R
47	B	*CIT*	*+*	R	R	R	S	S	R	R	R
498	B	*DHA*	*+*	R	R	S	R	R	R	R	R
474	C	*EBC*	*-*	R	R	S	S	S	R	S	S
74	C	*CIT*	*-*	S	S	S	S	S	R	S	S
485	C	*CIT*	*+*	R	R	R	R	S	R	R	R
434	C	*CIT*	*-*	R	R	S	S	S	R	S	R
427	C	*CIT*	*+*	R	R	R	S	S	R	S	R
396	D	*DHA*	*-*	R	R	S	R	S	R	S	S
59	D	*CIT*	*-*	S	R	R	S	S	R	S	S
448	D	*CIT*	*-*	S	R	S	S	S	R	R	R
410	D	*CIT*	*-*	R	R	R	S	S	R	S	R
206	D	*DHA*	*+*	R	R	R	R	S	R	S	R
27	D	*CIT*	*-*	S	R	R	S	S	R	S	I
263	D	*DHA*	*+*	R	S	R	S	S	R	S	R
40	D	*CIT*	*-*	S	S	S	S	S	R	S	S
179	E	*CIT*	*-*	S	S	S	S	S	R	S	S
18	E	*CIT*	*-*	S	S	R	S	S	R	S	S
46	F	*CIT*	*+*	R	R	R	R	S	R	S	R
22	F	*CIT*	*-*	R	S	S	I	S	R	S	R
64	F	*CIT*	*+*	R	R	R	S	S	R	R	R
174	F	*CIT*	*+*	R	R	R	S	S	R	S	R
180	Singleton	*CIT*	*+*	S	R	R	S	S	R	R	R

MLVA, Multi Locus VNTR Analysis; ESBL, Extended Spectrum Beta-Lactamase; CRO, Ceftriaxone; CIP, Ciprofloxacin; SXT, Trimethoprim/Sulfamethoxazole; TZP, Piperacillin-Tazobactam; MEM, Meropenem; AMC, Amoxicillin/clavulanic acid; GEN, Gentamicin; CAZ, Ceftazidime.

## Discussion

Plasmid-mediated AmpC β-lactamases have clinical significance due to causing serious challenges in the treatment procedure, drug resistance surveillance, epidemiological process, and infection management programs. In this context, the determination of the AmpC-producing isolates can greatly contribute to both surveillance and infection control for preventing nosocomial outbreaks and treatment failure (Saffar et al., 2016). β-Lactamase resistance is more prevalent in *Enterobacterales* compared to other gram-negative bacilli (Xu et al., 2015). Some factors can explain this condition: the extensive use of β-lactam antibiotics, in particular, broad-spectrum cephalosporins (Turnidge, 1998). No strategy is currently available to detect and confirm AmpC β-lactamases in clinical microbiology labs (Nikaido and Pagès, 2012). Nonetheless, the molecular evaluation of these isolates highlights its clinical detection. Thus, the molecular study of β-lactamases, in particular, AmpC type, is of crucial significance in terms of gene determinants and molecular epidemiology. Here, more than 54% (19.35) of *E. coli* isolates coproduced AmpC and ESBL and the remaining 46% of isolated *E. coli* showed only AmpC-producing features. It was previously reported that AmpCs β-lactamase is capable of hiding the second resistance phenotypes like ESBL (Nakano et al., 2017). As a result, the production of plasmid-mediated AmpC in an isolate may lead to false-negative results concerning ESBLs. These results can be helpful in epidemiological studies or infection management. The ESBL-suspected *E. coli* isolates must be tested for AmpC β-lactamases. In this research, screening tests on 16 *E. coli* isolates showed positive AmpC β-lactamase findings, meanwhile negative-ESBL results based on confirmation test (CAZ-CLA versus CAZ). About 40% (14.35) of AmpC-producing isolates exhibited resistance upon exposure to gentamicin. Moreover, 65.7% (23.35) and 74.2% (26.35) isolates were resistant against trimethoprim-sulfamethoxazole and ciprofloxacin, respectively.

Observing plasmids possessing AmpC-encoding genes next to other resistance genes to other antibiotics can be alarming for treatment setting (Hopkins et al., 2006). Based on antimicrobial susceptibility assays, AmpC-positive isolates exhibited a great resistance to cephalosporins when compared with their AmpC-negative counterparts, some of them showed resistance to aminoglycosides and quinolones (**Table 3**). This highlights the significance of detecting AmpC-producing isolates. This important issue should be considered by clinicians when using cephalosporins. The detection of the AmpC-producing isolates could influence antimicrobial therapy.

The AmpC-encoding genes such as *bla_FOX_*, *bla_CIT_*, and *bla_EBC_* have been found in previous works (Mansouri and Hoseini), while *bla_ACC_* class was not reported in any of them (Mansouri et al., 2009; Nahid Hoseini et al., 2017). *bla_FOX_* family showed the highest prevalence in these works. While *bla_MOX_* class was not detected in the Mansouri’s research, Hoseini reported the presence of *bla_FOX_* and *bla_CIT_*. Other studies in different countries (e.g. Egypt) indicated 57.7% Gram-negative AmpC-producing bacilli among which, 22 isolates carried *bla_MOX_*, *bla_FOX_*, and *bla_CIT_* families (Mansouri et al., 2009; Helmy and Wasfi, 2014; Nahid Hoseini et al., 2017). In Egypt, Helmy (Helmy and Wasfi, 2014) reported the *bla_CMY_* homologs as the most dominant gene (86.9%); *bla_DHA_* (21.7%), *bla_FOX_* (17.3%), *bla_EBC_* (13%), and *bla_MOX_* (13%) had the next ranks. Helmy’s study did not observe the *bla_ACC_* class. In this research, however, *bla_CIT_* had the highest frequency (73.7%) among the AmpC-encoding families; *bla_EBC_* (15.8%), *bla_FOX_* (10.5%), and *bla_DHA_* (10.5%) allocated the subsequent ranks, respectively. No members of *bla_ACC_* class were found. This enzyme could not be fully inhibited by cefoxitin (Nadjar et al., 2000; Helmy and Wasfi, 2014). Thean et al. (Tan et al., 2009) found that screening to find cefoxitin resistance has a lower sensitivity toward *bla_ACC_* family. Therefore, cefoxitin resistance could be exploited as a screening approach for the differentiation of *bla*_ACC_ enzymes.

The geographical region and the duration of the research also affected the prevalence and type of plasmid-mediated AmpCs (Ruppé et al., 2006; Adler et al., 2007). Numerous clinical microbiological laboratories fail in diagnosing the resistance mechanisms (Kashef et al., 2012). In the cases of simultaneous various antibiotics-resistance mechanisms, the phenotypic detection of AmpC enzymes could be very difficult; thus, molecular methods are recommended (Moland et al., 2008). According to our findings, AmpC β-lactamases can be related to false results of antimicrobial susceptibility tests for cephalosporins, therefore, the detection of AmpC-producing isolates is important for ESBL-negative isolates (Moland et al., 2008; Gupta et al., 2014).

The MLVA method was also employed to determine the genotypes of AmpC-producing *E. coli*. Three isolates were lost; hence, the remaining thirty-five AmpC-producing *E. coli* isolates were evaluated to indicate their clonal association by MLVA approach. MLVA showed thirty-one distinct patterns of AmpC-producing *E. coli* isolates which can be classified into six clusters and one singleton (Cutoff 80%). The singleton and MLVA type E isolates may be transmitted to the hospital by the patients, visitors, or medical staff as they had not been established in the hospital. MLVA Type B and MLVA Type D showed the highest prevalence and MLVA Type A, MLVA Type C, and MLVA Type F had the subsequent ranks, respectively. The mentioned MLVA types could be nosocomial infections; highlighting the necessity of evaluating MLVA types in the strains isolated from the patients and hospital staff. The two most prevalent MLVA types (Types A and B) exhibited two determinant genes of AmpC enzymes (*bla_CIT_*-*bla_EBC_* and *bla_CIT_*-*bla_FOX_*), whereas the other MLVA types only held one AmpC-encoding gene (**Table 1**). These results suggest the existence of the plasmids containing two genes in MLVA types A and B which can be transmitted by conjugating with other isolates. Thus, it is essential to identify these isolates and determine their transmission routes to establish a proper infection control strategy to prevent the release of resistance genes.

The determination of the frequency of AmpC-producing urinary *E. coli* isolates plays an important role in the antibiotic resistance and infection management programs in hospitals. The appearance of plasmid-mediated AmpC-producing *E. coli* can result in the spread of antibiotics resistance in the clinical settings. This dissemination can occur because of plasmid-mediated genes that may serve as the reservoir for antibiotic resistance. Therefore, the study of common gene determinants of AmpC resistance and its distribution in the bacterial species is of urgent necessity.

## Data Availability Statement

The raw data supporting the conclusions of this article will be made available by the authors, without undue reservation.

## Ethics Statement

The studies involving human participants were reviewed and approved by The Ethics Committee of Islamic Azad University in IRAN (registration number IR.IAU.PS.REC.1397.306). The patients/participants provided their written informed consent to participate in this study.

## Author Contributions

AD: study design and implementation and funding. SH, MR, MF and MD: scientific support. MF: supplier of laboratory site and equipment. MR: study management. All authors contributed to the article and approved the submitted version.

## Conflict of Interest

The authors declare that the research was conducted in the absence of any commercial or financial relationships that could be construed as a potential conflict of interest.
